# Selection of mitochondria in female germline cells: is Balbiani body implicated in this process?

**DOI:** 10.1007/s10815-017-1006-3

**Published:** 2017-07-28

**Authors:** Szczepan M. Bilinski, Malgorzata Kloc, Waclaw Tworzydlo

**Affiliations:** 1 Department of Developmental Biology and Invertebrate Morphology, Institute of Zoology and Biomedical Research, Jagiellonian University, Gronostajowa 9, 30-387 Krakow, Poland; 2 The Houston Methodist Research Institute and The Houston Methodist Hospital, 6670 Bertner Ave, Houston, TX 77030 USA

**Keywords:** Balbiani body, mtDNA bottleneck, Mitochondrial network, Selection of mitochondria, Oocyte

## Abstract

Early oocytes of nearly all animal species contain a transient organelle assemblage termed the Balbiani body. Structure and composition of this assemblage may vary even between closely related species. Despite this variability, the Balbiani body always comprises of numerous tightly clustered mitochondria and accumulations of nuage material. It has been suggested that the Balbiani body is an evolutionarily ancestral structure, which plays a role in various processes such as the localization of organelles and macromolecules to the germ plasm, lipidogenesis, as well as the selection/elimination of dysfunctional mitochondria from female germline cells. We suggest that the selection/elimination of mitochondria is a primary and evolutionarily ancestral function of Balbiani body, and that the other functions are secondary, evolutionarily derived additions. We propose a simple model explaining the role of the Balbiani body in the selection of mitochondria, i.e., in the mitochondrial DNA (mtDNA) bottleneck phenomenon.

## Introduction

In all animal species, mitochondria (containing molecules of mtDNA) are inherited from one generation to the next in a non-Mendelian manner, exclusively being passed on only by the mother. Several lines of evidence suggest that mitochondria overloaded with mutations in mtDNA are selectively eliminated from the growing oocytes [1–4]. Although, the mechanism of this purifying selection is far from being understood, it is widely accepted that it relies on the mtDNA bottleneck phenomenon [5–8], which involves an expansion of healthy mitochondria within the oocyte cytoplasm (ooplasm), selective directional transport of mitochondria to the germ plasm (located at the vegetal cortex in frogs and the posterior oocyte pole in holometabolous insects), and elimination of dysfunctional mitochondria by apoptosis of oocytes with excessive deleterious mtDNA mutations [1, 2, 4]. It has recently been proposed that the selection of mitochondria in developing oocytes is related to the formation and dispersion of the Balbiani body [3, 8, 9]. We will explore this idea and present a model explaining the role of the Balbiani body in the bottleneck phenomenon and elimination of dysfunctional mitochondria from the growing oocytes.

## Mitochondria and mitochondrial dynamics

Mitochondria are semi-autonomous organelles that contain their own genome (mtDNA) and protein synthesis machinery. The most important role of mitochondria is to supply cells with metabolic energy (ATP) generated by oxidative phosphorylation. Apart from generating ATP, oxidative phosphorylation produces reactive oxygen species (ROS), which predispose mtDNA to mutations. Gradual accumulation of these mutations leads, in turn, to numerous deleterious effects, including a loss of metabolic functions and decline in mitochondrial bioenergetic capacity. It is well established that these negative effects are counteracted by the process termed “mitochondrial dynamics” or “mitochondrial homeostasis.”

Mitochondrial dynamics involves two opposed processes: mitochondrial fusion and fission. Disruption of mitochondrial fusion prevents formation of mitochondrial networks and leads to maintaining mitochondria individuality, whereas obliterating fission results in the formation of extensive mitochondrial networks (hyperfused networks). Thus, the actual morphology of mitochondria in a differentiating or proliferating cell depends on a balance between mitochondrial fusion and fission (reviewed in [10, 11]). What is important, both fusion and fission are implicated in maintenance and inheritance of mtDNA: damaged mitochondria (containing mutated mtDNA) might be rescued by fusion or eliminated after fission, i.e., after separation from the network [12–15]. The fusion and fission are mediated by specific proteins belonging to the family of Dynamins. In humans, three main proteins involved in fusion are Optic atrophy 1 (OPA1) and the two mitofusins, MFN1 and MFN2. The major regulator of mitochondrial fission is a highly conserved Dynamin-related protein 1 (Drp1) (see [11] for a review).

Damaged mitochondria, separated from the network by fission, are subsequently eliminated by mitophagy (a specific type of authophagy that removes old and dysfunctional organelles). The mitophagy involves the cooperation of two proteins: the kinase PINK1 (PTEN-induced putative kinase 1) and cytosolic E3 ubiquitin ligase Parkin. The PINK1/Parkin system acts as a sensor for mitochondrial quality and is activated after the loss of the inner membrane potential of the mitochondria (see [16, 17] for a review). Interestingly, the mutations in PINK1 and Parkin genes have been identified as the genetic risk factors in familiar cases of Parkinson’s disease.

## Balbiani body: composition and suggested functions

Early oocytes of almost all studied animal species contain transient organelle assemblage called the Balbiani body (Bb) or “mitochondrial cloud”. Structure and composition of the Bb differ even in closely related species and are highly dynamic during subsequent stages of oogenesis. Despite this variability, the Bb always contains two essential elements: numerous tightly clustered mitochondria and accumulations of electron-dense granulo-fibrillar material, known as the nuage (molecular composition of nuage is discussed in [18–21]). The Bb may also comprise of Golgi complexes, elements of rough (coated with ribosomes) and smooth (lacking bound ribosomes) endoplasmic reticulum and centrioles [21, 22]. The composition of the Bb has been best described and understood in two model vertebrates: the frog, *Xenopus laevis*, and the fish, *Danio rerio*. In *Xenopus* oocytes, the Bb forms during early oogenesis by a gradual aggregation and multiplication of mitochondria around the pair of centrioles, i.e., the centrosome [22]. The stage I (previtellogenic) *Xenopus* oocyte contains fully developed spherical Bb located next to the oocyte nucleus and composed of thousands of mitochondria interspersed with accumulations of nuage, Golgi complexes, and elements of the rough endoplasmic reticulum (RER). The formation of Bb breaks the symmetry of oocyte cytoplasm and leads to its clear separation into animal (containing the oocyte nucleus) and vegetal (containing the Bb) hemispheres. At the same time, a future animal-vegetal axis of the embryo is established. During later stages of oogenesis, the Bb disperses and its constituents are transported toward the vegetal pole of the oocyte. Recent molecular studies have shown that the *Xenopus* Bb contains proteins and localized maternal RNAs implicated in the formation of the germ cell determinants (the germ plasm) (reviewed in [22]). The most up-to-date lists of molecular components of *Xenopus* Bb and Bb-derived germ plasm are tabulated in [23, 24]. In general, mRNA components can be grouped into three categories: (1) mRNAs encoding RNA-binding proteins, e.g., Nanos2 (Xcat2), Xdazl, and Hermes; (2) mRNAs encoding proteins involved in primordial germ cell migration; and (3) mRNAs implicated in recruitment of the germ plasm components such as Xpat and Xlsirts. Proteins identified in *Xenopus* Bb belong to various categories, such as translational repressors, anti-transposon factors, cell cycle regulators, and proteins involved in transport, as well as the localization of RNAs [24]. Although it remains unknown how the structural and molecular components of the frog and fish Bbs are assembled into such structurally intricate assemblage, it has been shown that two genes—*bucky ball* and *Xvelo* (in *Danio* and *Xenopus*, respectively)—are required for the Bb formation. Most recently, elegant molecular studies have indicated that *Xvelo* encodes a disordered protein with an N-terminal prion-like domain, and that this protein is an abundant constituent of the *Xenopus* Bb [25]. In the light of these results, it has been suggested that the frog Bb is formed by amyloid-like self-assembly of Xvelo, accompanied by a co-recruitment of mitochondria and RNAs [25]. It is important to add here that self-assembly of Xvelo, implicated in the formation of the Bb in frog oocytes, resembles the self-assembly of amyloid-β peptide engaged in the formation of amyloid plaques in Alzheimer’s diseased brain.

The Bb is also present in the oocytes of mammals, including mice and humans [26–28]. Ultrastructural studies have shown that mouse Bb is organized around the centrosome and consists of Golgi complexes surrounded by numerous aggregated mitochondria interspersed with elongated cisternae of the RER [27, 28]. Interestingly, it has been estimated that in mice oocytes, the number of mitochondria remaining in direct physical contact increases significantly (from 21 to 58%) during the formation of the Bb [29]. This previously omitted observation suggests that in mammals, as in insects (see section “Balbiani body: 3D organization”), mitochondria of the Bb may form a more or less extensive network.

Among invertebrates, the Bbs have been described in almost all investigated taxa. In some arthropods, e.g., spiders and myriapods, the Bbs are large, conspicuous, and persist in the ooplasm until the advanced stages of oogenesis [30–34]; in other species, the Bbs are relatively small and transient [35–37]. The most detailed and comprehensive description of the Bb morphology and morphogenesis in non-model invertebrates comes from the ultrastructural (EM) analyses of *Thermobia domestica* (firebrat) oocytes [9, 21, 37, 38]. This species has been selected as a “model” in our laboratory because of three reasons:

1. *T. domestica* belongs to the basal insect clade Zygentoma (silverfish), which suggests that in this species, ancestral mechanisms can operate during formation and dispersion of the Bb;
2. The Bb of this species is relatively small and consists of limited number of organelles that obviously simplifies analyses, especially at an EM level;
3. The oocytes and eggs of *T. domestica* are roughly spherical and do not comprise of any specialized region, e.g., the germ plasm, implying that the Bb in this basal insect is not involved in directional localization of mRNAs and/or organelles to certain oocyte region as it is in model species (frog or fish).

**Fig. 1:**
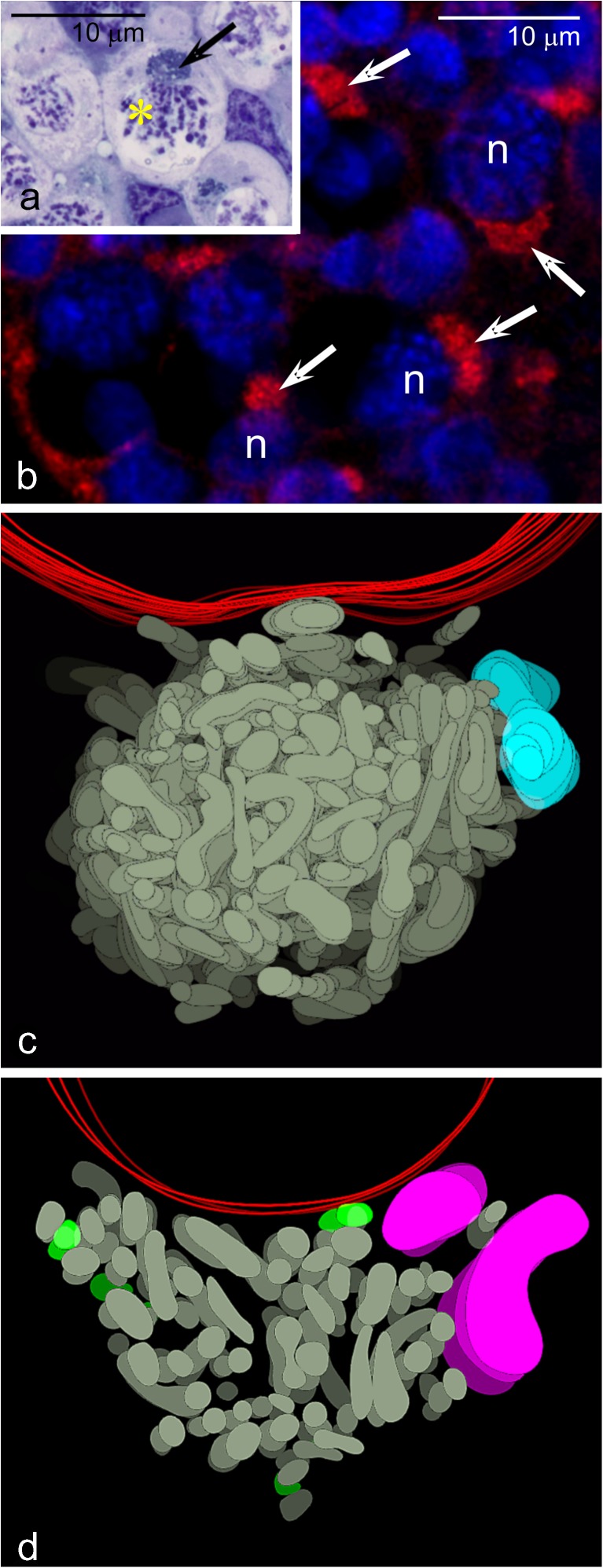
Balbiani body of *Thermobia domestica*. **a** Bouquet stage oocyte; Balbiani body (*arrow*) and chromosome bouquet (*yellow asterisk*); semithin section stained with methylene blue. **b** Fragment of the ovary incubated with MitoTracker, counterstained with Hoechst 33342. Highly positive Balbiani bodies (*white arrows*) of bouquet stage oocytes; oocyte nucleus (*n*). **c** Computer-aided reconstruction of the Balbiani body in the bouquet stage oocyte; mitochondrial network (*gray*), nuage accumulation (*blue*), nuclear envelope (*red*). For this reconstruction, full series of 15 ultrathin sections has been used. **d** Partial reconstruction of the Balbiani body in bouquet stage oocyte; mitochondrial network (*gray*), Golgi complex (*magenta*), degenerating mitochondria (*green*). For this reconstruction, only three serial sections have been used. For the clarity of the reconstructions, colors of subsequent “levels” representing subsequent sections have been depicted as successively darker. **a**, **c**, and **d** reprinted from [9]

Our studies have shown that in *T. domestica*, the Bb starts to form in premeiotic germline cells (the cystoblasts, Cbs), soon after asymmetric division of the female germline stem cell [37, 39]. EM studies have shown that at this initial stage, the Cb mitochondria are already differentiated into two characteristic subpopulations: elongated or bifurcated and small rod-like. Interestingly, the first subpopulation has been observed in contact with the nuage material [9]. The Bbs of early meiotic oocytes are much larger and compact. These observations have led to an assumption that during morphogenesis of the Bb, its mitochondria quickly multiply, and that this process is initiated by the nuage [9]. Furthermore, analysis of serial sections has revealed that at the so-called bouquet stage of meiotic prophase, the Bb is invariably located next to the segment of nuclear envelope where the telomeres of the bouquet chromosomes are attached (Fig. 1a). It is interesting to note that similar arrangement of the Bb in relation to bouquet chromosomes has been also noted in such phylogenetically distant species as *X. laevis* [40], *D. rerio* [41], and mice (unpublished data) suggesting that such association must be an old and conserved trait of oogenesis. The Bb of *T. domestica* disperses relatively early—at the onset of the previtellogenic growth [9, 37] (Fig. 5b). As dispersion progresses, the Bb mitochondria become highly elongated (filiform) and gradually populate the entire ooplasm [9].

Until recently, three principal functions have been ascribed to the Bb:

- delivery of germ cell determinants and localized mRNAs to the oocyte vegetal cortex/germ plasm in *Xenopus* oocytes [42–45],
- transport of the mitochondria to the germ plasm (the vegetal cortex or posterior oocyte pole in *Xenopus* and *Drosophila*, respectively) [35, 42, 46],
- participation in the formation of lipid droplets (in certain spiders) [34].

It has been suggested, in this context, that the Bb represents an evolutionarily ancestral structure involved in the localization and/or enrichment of maternal macromolecules (RNAs) and organelles (mitochondria) to certain oocyte regions (reviewed in [21]).

## Balbiani body: 3D organization

**Fig. 2:**
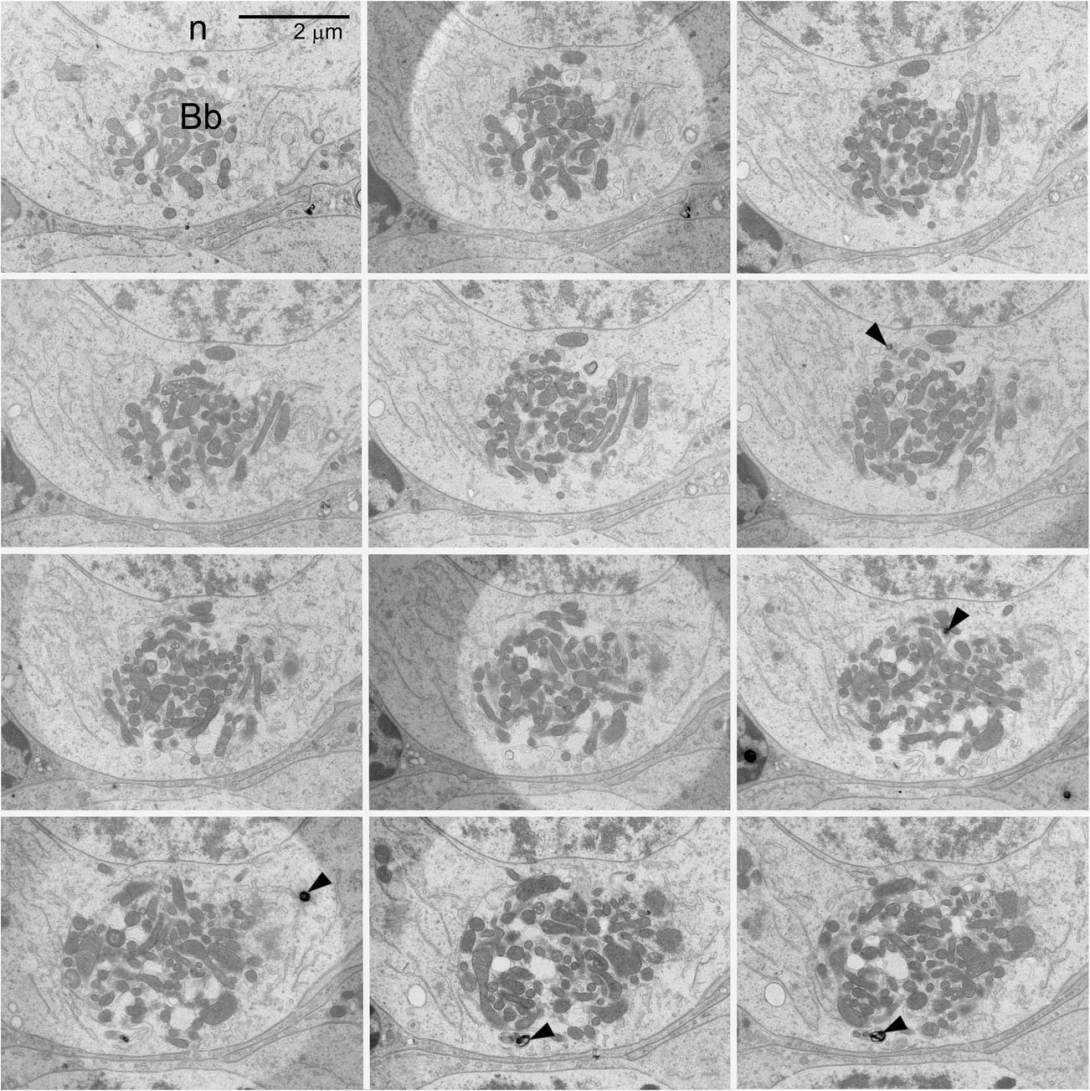
Series of 12 ultrathin sections through the central part of *Thermobia* Balbiani body in bouquet stage oocyte; Balbiani body (*Bb*), oocyte nucleus (*n*), degenerating mitochondria (*arrowheads*)

Until recently, the morphology and ultrastructure of Bb have been studied using single, often incidental thin or ultrathin sections. In consequence, despite relatively numerous analyses, the 3-dimensional (3D) organization of the Bb and the exact relationships between its constituents remain contentious. In this context, we have reconstructed 3D organization of the *Thermobia* Bb at the EM level using serial ultrathin (200 nm) sections (Fig. 2) and computer-aided imaging [9]. Quite unexpectedly, we have unraveled that at the bouquet stage, the core part of the *Thermobia* Bb is occupied by an extensive mitochondrial network (Fig. 1c, d), which is surrounded by nuage accumulations, Golgi complexes, RER elements, and individual rod-like mitochondria (Fig. 1c, d). The latter organelles were often morphologically altered showing signs of degeneration (Fig. 1d, green; Fig. 2, arrowheads). This led to speculation that in *Thermobia* oocytes, damaged mitochondria are separated from the mitochondrial network of the Bb and are subsequently eliminated in the ooplasm by mitophagy [9].

**Fig. 3:**
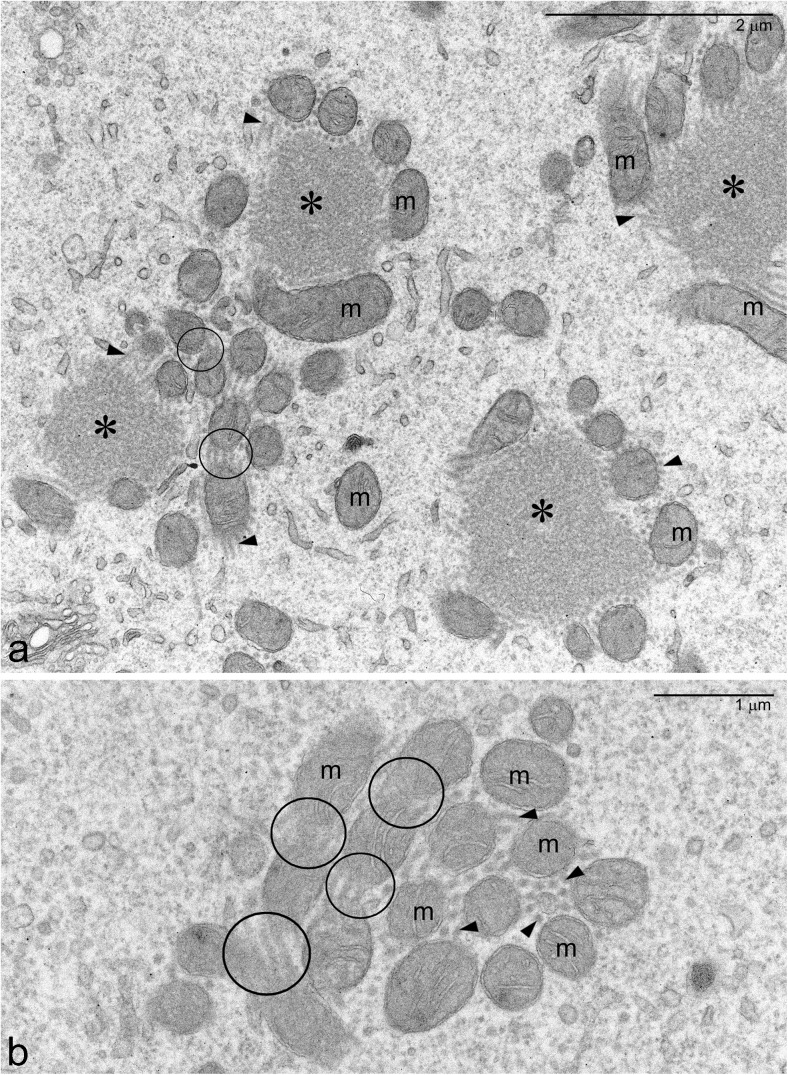
Balbiani body of *Metrioptera brachyptera*. **a**, **b** Mitochondria (*m*) clustered around large nuage accumulations (*asterisks*) and surrounded by nuage filaments (*arrowheads*); Note that some mitochondria remain in direct contact (*encircled*)

To check whether the Bbs of other insect species contain mitochondrial networks, we have analyzed the ultrastructure of developing oocytes of *Metrioptera brachyptera* (grasshopper). Our unpublished results indicate that the grasshopper Bb is not as compact as that of *Thermobia*, but rather consists of several isolated groups (islands) of mitochondria clustered around conspicuous nuage accumulations, and tightly entwined by thick nuage filaments (Fig. 3a, b). Even though the precise reconstruction of 3D organization of the *Metrioptera* Bb has not yet been performed, careful analysis of single micrographs unambiguously shows that mitochondria of a given group (island) are interconnected and form a mitochondrial network or “micro-network” (Fig. 3a, b).

**Fig. 4:**
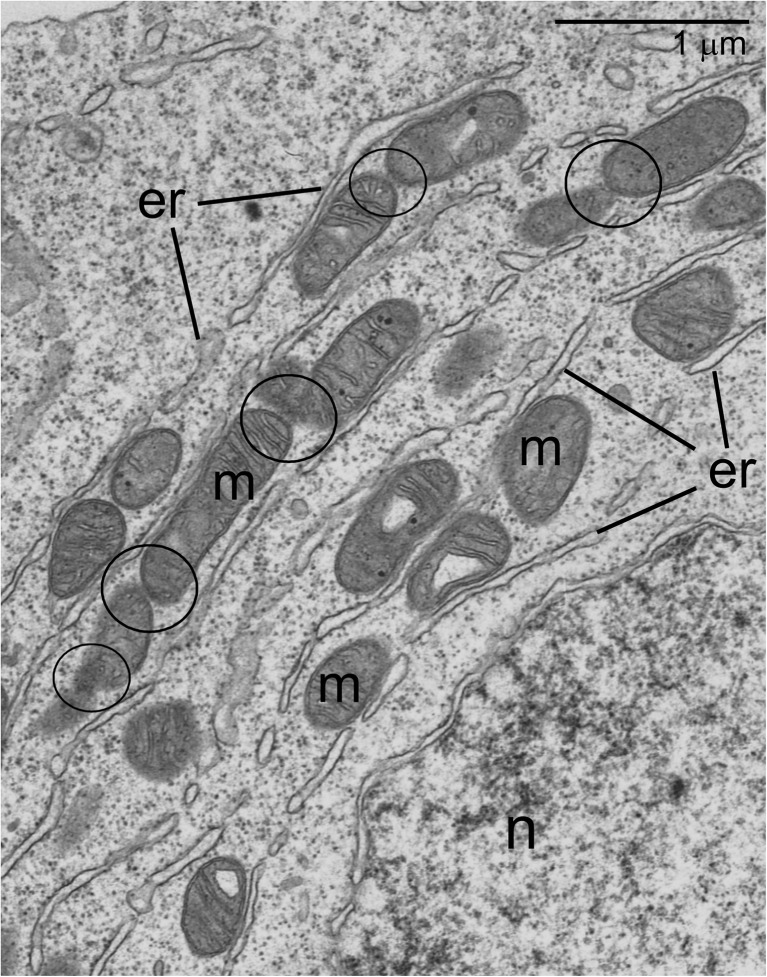
Peripheral part of mouse Balbiani body; endoplasmic reticulum (*er*), mitochondria (*m*), oocyte nucleus (*n*); Note that some mitochondria remain in direct contact (*encircled*). (Courtesy of Dr. M. Jaglarz, Institute of Zoology and Biomedical Research, Jagiellonian University, Poland)

The 3D organization of the Bb in murine oocytes is still not fully clarified. However, partial reconstructions of the Bb ultrastructure [28] as well as reinterpretation of certain microphotographs published elsewhere (e.g., Fig. 5B and F in [29]; Fig. 1F in [27]; Fig. 7B in [28]) strongly suggest that also in this species, the Bb mitochondria are interconnected and form a network (Fig. 4). This assumption is additionally reinforced by the observation that, at the onset of formation of murine Bb, the mitochondria not only increase in number but also become much more aggregated (clustered) [29] (see also section “Balbiani body: composition and suggested functions”).

## Balbiani body: physiological state of Bb mitochondria

Since the pioneering work of Tourte and colleagues [47], it has been well established that the Bb mitochondria physiologically differ from those located outside the Bb [46–51]. Furthermore, more recent analyses involving incubation of oocytes in media supplemented with fluorescent probes (MitoTracker, JC-1) have indicated that the mitochondria constituting the Bb show higher inner membrane potential [9, 50, 52] (Fig. 1b). These observations have led to an assumption that only high membrane potential mitochondria are recruited to the Bb [3, 8] and that mitochondria with lowered membrane potential (i.e., overloaded with mutations in mtDNA) are excluded from the Bb and subsequently eliminated by mitophagy [9]. Thus, only the most fit and active mitochondria are transmitted to the next generation [3, 4, 8, 53].

## Conclusions: a model explaining selection of mitochondria in oocytes

Animal ovaries fall into two morphologically and physiologically different categories: panoistic and meroistic. This clearly dichotomous split should be taken into consideration while interpreting data summarized in this review. Therefore, we will start this section with a very short description of two basic ovarian categories. For comprehensive and detailed description of all ovarian types and subtypes, see [39, 54–57].

Panoistic ovaries are characteristics for fish, amphibians, and the majority of invertebrates, e.g., spiders, higher crustaceans, and hemimetabolous insects. In this ovary type, all female germline cells (with exception of degenerating ones) develop into fertilizable egg cells. This implies that all the mitochondria of a given egg are descendants of the mitochondria of a single oogonial cell. Merositic ovaries have been found in annelids, lower crustaceans, holometabolous insects, including the fruit fly and certain lizards. Here, the oocytes develop within syncytial cysts (clusters) of sibling germline cells [55]. In each cluster, usually only one cell differentiates into the oocyte, whereas the others become supporting nurse cells. During oogenesis, the growing oocyte receives organelles, including mitochondria, and various macromolecules from associated nurse cells. Interestingly, it has been recently shown that also murine ovaries are meroistic; murine oocytes develop in syncytial cysts and acquire organelles from sister “nurse” cells [58, 59]. This, in turn, implies that in mice as well as in other species with meroistic ovaries, the mitochondria of a given egg cell may originate from several interconnected sibling cells.

**Fig. 5:**
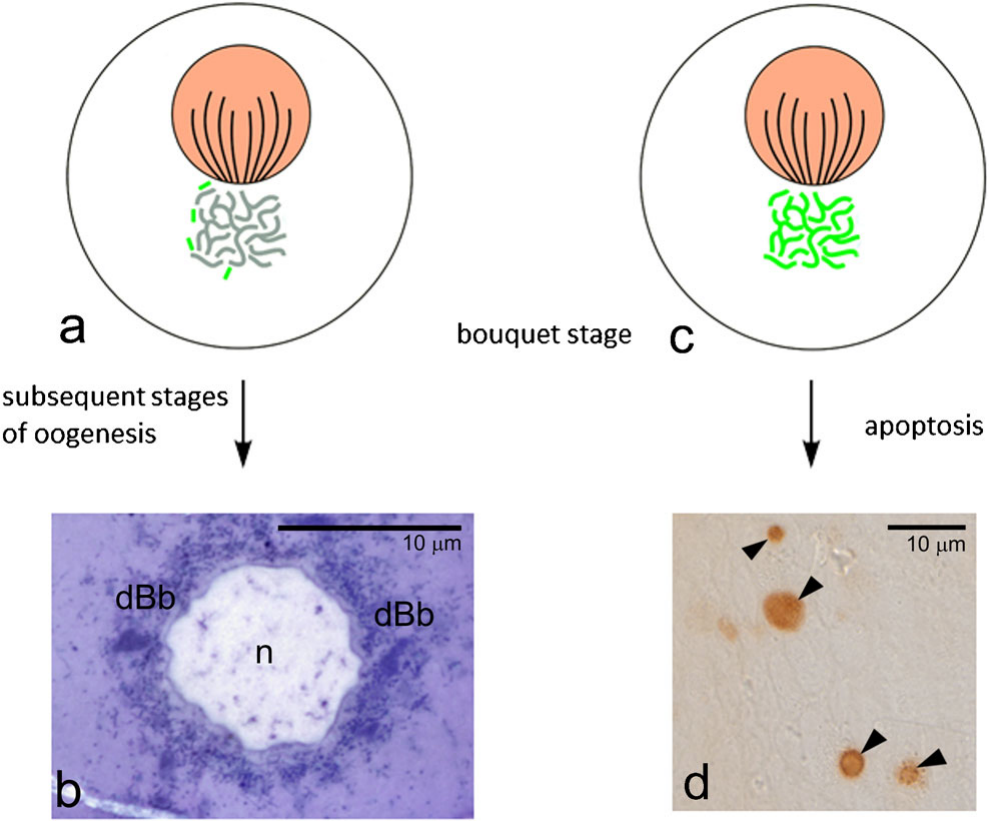
The second step of mitochondria selection model. **a** Bouquet stage oocyte with the Balbiani body composed of selected “healthy” mitochondria (*gray*); mitochondria separated from the Balbiani body network and degenerating in the cytoplasm (*green*), oocyte nucleus with bouquet chromosomes (*pink*). **b** When such oocytes (*arrow*) continue oogenesis, their Balbiani bodies disperse in the ooplasm; oocyte nucleus (*n*), dispersing Balbiani body (*dBb*). *Thermonbia domestica*, semithin section stained with methylene blue. **c** Bouquet stage oocyte that has not successfully “cleared off” mtDNA mutations; its Balbiani body comprises only dysfunctional mitochondria (*green*), oocyte nucleus with bouquet chromosomes (*pink*). **d** Oocytes with dysfunctional mitochondria (*arrow*) are eliminated by apoptosis. *Thermobia domestica*, whole mount preparation stained with TUNEL assay; apoptotic cells are indicated by *arrowheads*. **b** and **d** reprinted from [9]

The involvement of the Bb in the selection of the mitochondria in developing oocytes has been already suggested [3, 8, 9]. Critical review of all available data supports this idea and points out that the Bb might be an important player in the bottleneck phenomenon. We suggest the following scenario of the Bb participation in mitochondria selection:

- The Bb forms around the centrosome (vertebrates) or nuage (insects). At this early stage of formation, the Bb mitochondria are not specifically selected, but instead they are chosen randomly. Thus, the Bbs of sibling germline cells stochastically receive mitochondria in various functional (physiological) states.
- As oogenesis progresses, the Bb mitochondria multiply and fuse forming an extensive hyperfused network.
- Dysfunctional mitochondria (containing mutated mtDNA) are separated from the network and eliminated by mitophagy.
- After dispersion of the Bb (Fig. 5b), healthy selected mitochondria can populate the whole ooplasm (in *Thermobia*) or they are transferred to the germ plasm, i.e., the vegetal cortex in *Xenopus* or the posterior oocyte pole in the fruit fly and other holometabolous insects.

The proposed scenario agrees well with existing theoretical models of the mitochondrial quality control assuming that fusion, fission, and mitophagy together increase mitochondrial functionality (reviewed in [60]). It is obvious that the proposed scenario should be slightly modified in the case of polytrophic ovaries, especially when mitochondria from several sibling cells participate in the formation of the Bb [35, 58, 59].

It is well established that in the ovaries of almost all investigated species, the large proportion of germline cells (up to 18% in *Thermobia*, 70% in mammals) are eliminated via apoptosis (Fig. 5d) (see [61, 62] for further reading). In this context, we suggest that the selection of the mitochondria in the female germline occurs in two steps. The first step operates in the cytoplasm of germline cells and involves selection of mitochondria within the Bb. In the second step, the germline cells that have not been successfully “cleared of,” deleterious mtDNA mutations are removed by apoptosis (Fig. 5). This two-step model agrees well with earlier suggestions that in mammalian ovaries, apoptosis of germline cells is induced by oxidative stress and that mitochondria overloaded with severe mutations in mtDNA produce more ROS and thus are preferentially eliminated [1, 62].

## Outlook

The accumulated data clearly indicate that in different animal lineages the Bb might be involved in various cellular processes. Among these are selection/elimination of defective germline mitochondria carrying mtDNA mutations, directional transport of organelles and macromolecules to the germ plasm, and formation of lipid droplets. The fact that the Bbs are present in all animal oocytes, regardless of whether they contain the germ plasm or not indicates that mitochondrial selection/elimination represents a primary and ancestral role for the Bb, whereas the other two functions are secondary and evolutionarily novel—having been adopted during the evolution of certain animal taxa. Interestingly, the directional transport function evolved in groups characterized by the preformation of the germ plasm (germline determinants), while the lipidogenesis function emerged only in a single arthropod lineage.

This proposed idea will require further investigation for its verification using both model and non-model species. Modern techniques of 3D imaging, such as EM tomography and serial block-face scanning electron microscopy (SBEM) [63, 64] would simplify and speed up this scientific inquiry. Molecular and genetic studies will be also crucial for justifying the suggested involvement of the Bb in the mtDNA bottleneck in mammalian and non-mammalian oocytes.
